# Generation of KCL024 research grade human embryonic stem cell line carrying a mutation in *NF1* gene

**DOI:** 10.1016/j.scr.2016.01.010

**Published:** 2016-03

**Authors:** Heema Hewitson, Victoria Wood, Neli Kadeva, Glenda Cornwell, Stefano Codognotto, Emma Stephenson, Dusko Ilic

**Affiliations:** Stem Cell Laboratories, Division of Women's Health, Faculty of Life Sciences and Medicine, King's College London and Assisted Conception Unit, Guys' Hospital, London, United Kingdom

## Abstract

The KCL024 human embryonic stem cell line was derived from an embryo donated for research that carried an autosomal dominant mutation in the *NF1* gene encoding neurofibromin (c.3739–3742 ∆ TTTG). Mutations in this gene have been linked to neurofibromatosis type 1, juvenile myelomonocytic leukemia and Watson syndrome. The ICM was isolated using laser microsurgery and plated on γ-irradiated human foreskin fibroblasts. Both the derivation and cell line propagation were performed in an animal product-free environment. Pluripotent state and differentiation potential were confirmed by in vitro assays.

## Resource table

**Table:** 

Name of stem cell line	KCL024
Institution	King's College London, London UK
Derivation team	Neli Kadeva, Victoria Wood, Glenda Cornwell, Stefano Codognotto, Emma Stephenson
Contact person and email	Dusko Ilic, email: dusko.ilic@kcl.ac.uk
Date archived/stock date	Mar. 31, 2011
Type of resource	Biological reagent: cell line
Sub-type	Human pluripotent stem cell line
Origin	Human embryo
Key marker expression	Pluripotent stem cell markers: NANOG, OCT4, TRA-1-60, TRA-1-81, alkaline phosphatase (AP) activity
Authentication	Identity and purity of line confirmed
Link to related literature (direct URL links and full references)	1) Ilic, D., Stephenson, E., Wood, V., Jacquet, L., Stevenson, D., Petrova, A., Kadeva, N., Codognotto, S., Patel, H., Semple, M., Cornwell, G., Ogilvie, C., Braude, P., 2012. Derivation and feeder-free propagation of human embryonic stem cells under xeno-free conditions. Cytotherapy. 14 (1), 122–128. doi: 10.3109/14653249.2011.623692 http://www.ncbi.nlm.nih.gov/pubmed/22029654 2) Stephenson, E., Jacquet, L., Miere, C., Wood, V., Kadeva, N., Cornwell, G., Codognotto, S., Dajani, Y., Braude, P., Ilic, D., 2012. Derivation and propagation of human embryonic stem cell lines from frozen embryos in an animal product-free environment. Nat. Protoc. 7 (7), 1366–1381. doi: 10.1038/nprot.2012.080 http://www.ncbi.nlm.nih.gov/pubmed/22722371
Information in public databases	KCL024 is a National Institutes of Health (NIH) registered hESC line NIH Registration Number: 0220 NIH Approval Number: NIHhESC-13-0220 http://grants.nih.gov/stem_cells/registry/current.htm?id=660
Ethics	The hESC line KCL024 is derived under license from the UK Human Fertilisation and Embryology Authority (research license numbers: R0075 and R0133) and also has local ethical approval (UK National Health Service Research Ethics Committee Reference: 06/Q0702/90). Informed consent was obtained from all subjects and the experiments conformed to the principles set out in the WMA Declaration of Helsinki and the NIH Belmont Report. No financial inducements are offered for donation.

## Resource details

**Table:** 

Consent signed	Oct. 28, 2010
Embryo used	Mar. 03, 2011
UK Stem Cell Bank Deposit Approval	Dec. 01, 2011 Reference: SCSC11-48
Sex	ND
Grade	Research
Disease status (Fig 1)	Autosomal dominant mutation in the *NF1* gene encoding neurofibromin (c.3739–3742 ∆ TTTG)
Karyotype (aCGH)	ND
DNA fingerprint	ND
HLA typing	HLA-A: 11,32; -B: 35,55; -C 03,15; DRB1: 04,11
Viability testing	Pass
Pluripotent markers (immunostaining) (Fig 2)	NANOG, OCT4, TRA-1-60, TRA-1-81, AP activity
Three germ layers differentiation in vitro (immunostaining) (Fig 3)	Endoderm: AFP (α-fetoprotein) Ectoderm: TUBB3 (tubulin, β3 class III) Mesoderm: ACTA2 (actin, α2, smooth muscle)
Sibling lines available	KCL025

ND, not determined.

We generated KCL024 clinical grade hESC line following protocols, established previously (Ilic et al., 2012, Stephenson et al., 2012). The expression of the pluripotency markers was tested after freeze/thaw cycle. Differentiation potential into three germ layers was verified in vitro.

## Materials and methods

### Consenting process

We distribute Patient Information Sheet (PIS) and consent form to the in vitro fertilization (IVF) patients if they opted to donate to research embryos that were stored for 5 or 10 years. They mail signed consent back to us and that might be months after the PIS and consent were mailed to them. If in the meantime new versions of PIS/consent are implemented, we do not send these to the patients or ask them to re-sign; the whole process is done with the version that was given them initially. The PIS/consent documents (PGD-V.8) were created on Jul. 01, 2010. HFEA Code of Practice that was in effect at the time of document creation: Edition 8 — R.2 (http://www.hfea.gov.uk/2999.html). The donor couple signed the consent on Oct. 28, 2010. HFEA Code of Practice that was in effect at the time of donor signature: Edition 8 — R.2. HFEA Code of Practice Edition 8 — R.2 was in effect 07 Apr. 2010–Apr. 06, 2011.

### Embryo culture and micromanipulation

Embryo culture and laser-assisted dissection of inner cell mass (ICM) were carried out as previously described in details (Ilic et al., 2012, Stephenson et al., 2012). The cellular area containing the ICM was then washed and transferred to plates containing mitotically inactivated human neonatal foreskin fibroblasts (HFF).

### Cell culture

ICM plated on mitotically inactivated HFF were cultured as described (Ilic et al., 2012, Stephenson et al., 2012). TE cells were removed mechanically from outgrowth (Ilic et al., 2007, Ilic et al., 2010). hESC colonies were expanded and cryopreserved at the third passage.

### Viability test

Straws with the earliest frozen passage (p.2–3) are thawed and new colonies are counted three days later. These colonies are then expanded up to passage 8, at which point cells were part frozen and part subjected to standard battery of tests (pluripotency markers, in vitro and in vivo differentiation capability, genetics, sterility, mycoplasma).

### Pluripotency markers

Pluripotency was assessed using two different techniques: enzymatic activity assay [alkaline phosphatase (AP) assay] and immunostaining as described (Ilic et al., 2012, Stephenson et al., 2012).

### Differentiation

Spontaneous differentiation into three germ layers was assessed in vitro as described (Ilic et al., 2012, Stephenson et al., 2012, Petrova et al., 2014).

### HLA typing

HLA-A, -B and -DRB1 typing was performed with a PCR sequence-specific oligonucleotide probe (SSOP; Luminex, Austin, TX, USA) hybridization protocol at the certified Clinical Transplantation Laboratory, Guy's and St. Thomas' NHS Foundation Trust and Serco Plc. (GSTS) Pathology (Guy's Hospital, London, UK) as described (Jacquet et al., 2013).

## Author disclosure statement

There are no competing financial interests in this study.

## Figures and Tables

**Fig. 1:**
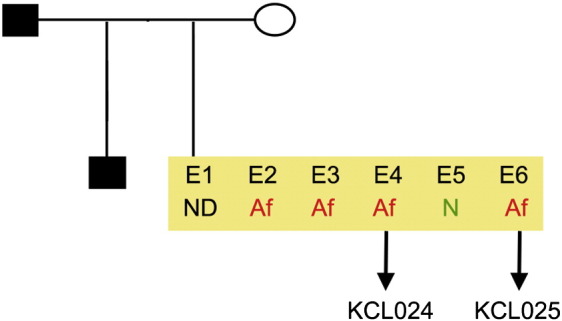
Genetic pedigree tree. Male donor was carrying an autosomal dominant mutation c.3739–3742 ∆ TTTG in the *NF1* gene. The couple undergoing IVF and prenatal genetic diagnosis had 6 embryos in this particular cycle. Embryos carrying the mutation in the *NF1* gene were donated for research. We derived two hESC lines: KCL024 and KCL025.

**Fig. 2:**
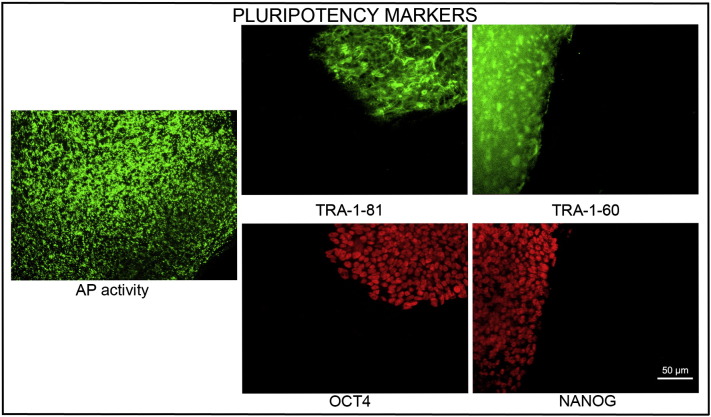
Expression of pluripotency markers. Pluripotency is confirmed by immunostaining (Oct4, Nanog, TRA-1-60, TRA-1-81) and alkaline phosphatase (AP) activity assay. Scale bar, 50 μm.

**Fig. 3:**
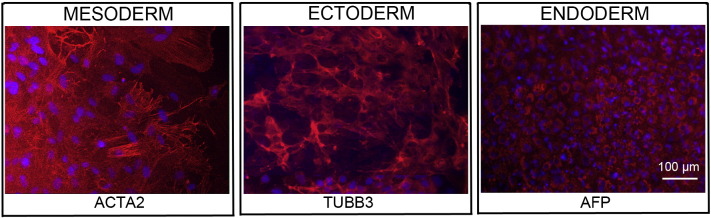
Differentiation of three germ layers in vitro is confirmed by detection of markers: smooth muscle actin (ACTA2, red) for mesoderm, β-III tubulin (TUBB3, red) for ectoderm and α-fetoprotein (AFP, red) for endoderm. Nuclei are visualized with Hoechst 33342 (blue). Scale bar, 100 μm.
